# Prognostic and Therapeutic Implications of Alamandine Receptor MrgD Expression in Clear Cell Renal Cell Carcinoma with Development of Metastatic Disease

**DOI:** 10.3390/biom15030387

**Published:** 2025-03-07

**Authors:** Gorka Larrinaga, Jon Danel Solano-Iturri, Inés Arrieta-Aguirre, Asier Valdivia, David Lecumberri, Ane Miren Iturregui, Charles H. Lawrie, María Armesto, Juan F. Dorado, Caroline E. Nunes-Xavier, Rafael Pulido, José I. López, Javier C. Angulo

**Affiliations:** 1 Department of Nursing, Faculty of Medicine and Nursing, University of the Basque Country (UPV/EHU), 48940 Leioa, Spain; ines.arrieta@ehu.eus; 2 Department of Physiology, Faculty of Medicine and Nursing, University of the Basque Country (UPV/EHU), 48940 Leioa, Spain; 3 Biobizkaia Health Research Institute, 48903 Barakaldo, Spain; jondanel.solanoiturri@osakidetza.eus (J.D.S.-I.); carolineelisabeth.nunes-xavier@bio-bizkaia.eus (C.E.N.-X.); rpulidomurillo@gmail.com (R.P.); joseignacio.lopez@biocrucesbizkaia.org (J.I.L.); 4 Pathology Department, Cruces University Hospital, 48903 Barakaldo, Spain; 5 Department of Cellular Biology and Histology, Faculty of Medicine and Nursing, University of the Basque Country (UPV/EHU), 48940 Leioa, Spain; asier.valdivia@ehu.eus; 6 Department of Urology, Cruces University Hospital, 48903 Barakaldo, Spain; david.lecumberricastanos@osakidetza.eus (D.L.); anemiren.iturreguidelpozo@osakidetza.eus (A.M.I.); 7 Molecular Oncology Group, Biogipuzkoa Health Research Institute, 20014 San Sebastián, Spain; charles.lawrie@bio-gipuzkoa.eus (C.H.L.); maria.armestoalvarez@bio-gipuzkoa.eus (M.A.); 8 IKERBASQUE, Basque Foundation for Science, 48009 Bilbao, Spain; 9 Radcliffe Department of Medicine, University of Oxford, Oxford OX3 9DU, UK; 10 Sino-Swiss Institute of Advanced Technology (SSIAT), Shanghai University, Shanghai 201800, China; 11 PeRTICA Statistical Solutions, Pl. Constitución, 2, 28943 Fuenlabrada, Spain; jfdorado@pertica.es; 12 Department of Tumor Biology, Institute for Cancer Research, Oslo University Hospital Radiumhospitalet, 0310 Oslo, Norway; 13 Clinical Department, Faculty of Medical Sciences, European University of Madrid, 28905 Getafe, Spain; javier.angulo@universidadeuropea.es

**Keywords:** clear cell renal cell carcinoma, metastatic, prognostic, tyrosin kinase inhibitors, biomarker, renin–angiotensin system, MrgD, Alamandine, immunohistochemistry

## Abstract

Despite advances in the management of advanced clear cell renal cell carcinoma (ccRCC), robust biomarkers for prognosis and therapeutic response prediction remain elusive. Dysregulation of the intrarenal renin–angiotensin system (RAS) has been implicated in renal carcinogenesis but little explored, particularly regarding biomarker discovery and therapeutic innovation. Consequently, this study investigates the immunohistochemical expression and clinical relevance of the Mas-related G-protein-coupled receptor D (MrgD) in patients with ccRCC who developed metastatic disease (mccRCC). A cohort of 132 patients treated between 2008 and 2018 with nephrectomy and tyrosine kinase inhibitor (TKI)-based sequential therapy was analyzed. Treatment response was assessed using both the MASS and RECIST scoring systems. High MrgD expression in primary tumors was significantly associated with larger size, advanced stage, higher histological grade, and worse overall survival. Among 81 patients with metachronous metastases, high MrgD expression independently predicted shorter disease-free survival. High MrgD staining intensity correlated with poorer TKI responses in first-line therapy but improved outcomes with second-line mTORC1 inhibitors. These findings suggest that MrgD may be a useful biomarker of RAS linked to tumor aggressiveness in ccRCC. MrgD holds potential for identifying high-risk patients and guiding treatment selection in advanced disease. Further research is needed to unlock its clinical potential.

## 1. Introduction

Clear cell renal cell carcinoma (ccRCC) is the most common subtype of kidney cancer, accounting for 70–80% of all renal malignancies [1]. It remains a significant global health burden, with over 430,000 new cases of renal cancer diagnosed annually worldwide and more than 180,000 related deaths in 2020 [2]. The disease’s impact is heightened by its tendency to present at advanced stages; approximately 30% of patients have metastatic disease at diagnosis, and another 30% with localized disease will progress to metastatic stages over time [3,4]. Despite advances in detection and surgical approaches, mccRCC remains a major clinical challenge, with 5-year survival rates under 15% in cases with widespread metastases [5].

The treatment of advanced ccRCC has seen significant improvement over the past years, driven by targeted therapies with tyrosine kinase inhibitors (TKIs) and the introduction of immune checkpoint inhibitors (ICIs) [6,7]. Combination therapies, such as ICI–ICI or ICI–TKIs, have emerged as first-line treatments for advanced disease, tailored according to patient risk profiles [6,7]. However, despite these innovations, the benefits are often heterogeneous, and a subset of patients shows resistance or limited response to current therapies.

This variability perpetuates the ongoing debate over the optimal implementations of available therapies in metastatic disease as well as the clinical relevance of current prognostic tools [1,5]. Current systems such as the International Metastatic Renal Cell Carcinoma Database Consortium (IMDC) risk classification system [8] can guide therapeutic decisions but lack molecular precision [5]. Efforts to identify reliable biomarkers capable of predicting prognosis and response to targeted or immune-based therapies have yielded many promising candidates; however, none have translated into routine clinical practice [9].

The renin–angiotensin system (RAS) has emerged as a promising area of research, offering novel opportunities for both biomarker development and therapeutic innovation in renal cancer [10,11,12,13,14,15,16]. The RAS is well known for its endocrine roles in regulating renal and cardiovascular physiology with local RASs exerting both paracrine and autocrine effects regulating long-term physiological phenomena in the kidney [17,18]. Specifically, the angiotensin-converting enzyme (ACE)/angiotensin II (Ang-II)/angiotensin type 1 receptor (AT1R) axis promotes cell growth, angiogenesis, and tissue repair, whereas the ACE2/angiotensin 1–7 (Ang 1–7)/MAS receptor (MASR) axis, often referred to as the alternative RAS [19], acts as a counter-regulatory mechanism [17,18,19].

Imbalances favoring the ACE/Ang-II/AT1R axis contribute to renal cell proliferation, inflammation, and fibrosis. This discovery has deepened our understanding of the therapeutic efficacy of ACE inhibitors (ACEis) and AT1R blockers (ARBs) in slowing the progression of non-neoplastic kidney diseases [17,19] and have broadened the scope of RAS research into renal carcinogenesis [10,13,14]. Notably, multiple studies have demonstrated that the use of these RAS inhibitors (RASis) improves responses to TKIS and ICI therapy and is associated with better outcomes for mccRCC patients [11,12,15,16]. Moreover, preclinical research has highlighted the pro-tumorigenic effects of the ACE/Ang-II/AT1R axis in renal tumors [10,13,14,20,21,22]. However, the role of the alternative RAS pathways in renal carcinogenesis remains less studied, with available data presenting often contradictory findings [23,24,25].

Alamandine (ALA), a recently discovered peptide of the alternative RAS [26], binds to the Mas-related G-protein-coupled receptor D (MrgD), exerting effects analogous to the Ang 1–7/MASR axis, including antifibrotic and antiproliferative actions in local contexts [19,26,27,28]. Despite its potential relevance, studies exploring the expression of MrgD in tumor tissues and the role of the ALA/MrgD axis in cancer progression are limited [29,30,31,32].

In the context of renal neoplasms, we recently demonstrated that MrgD is expressed in several renal tumor subtypes. Moreover, ccRCC patients with higher MrgD expression exhibited a more aggressive clinical behavior and were associated with worse patient survival [32]. Our pilot study focused on early ccRCCs, although a small subgroup of patients developed metastases and was treated with first-line TKIs, showing a poorer response when tumors had elevated MrgD expression [32].

In order to expand these findings to advanced-stage ccRCC, the current study analyzed the immunohistochemical (IHC) expression of MrgD in a cohort of metastatic ccRCC (mccRCC) patients. This study aimed to achieve three objectives: (1) to validate the association of MrgD expression with pathological parameters of tumor aggressiveness, including histological grade, tumor size, and local and distant invasion; (2) to determine whether MrgD expression in primary tumors predicts disease recurrence and overall survival; and (3) to explore its relationship with therapeutic responses across different treatment lines.

## 2. Materials and Methods

### 2.1. Patients

This study included patients with mccRCC treated between 2008 and 2018 at two tertiary reference centers, Donostia and Cruces University Hospitals in the Basque Country (Spain) [33]. This study was approved by the Institutional Ethics Committee (CEIm Euskadi, approval number PI2015059X). All patients underwent radical nephrectomy, which confirmed the histopathological diagnosis of ccRCC. Synchronous or metachronous metastases were diagnosed through imaging studies, often confirmed by biopsy or, in some cases, by surgical resection. Tumor classification at the time of nephrectomy followed the American Joint Committee on Cancer (AJCC) and the National Comprehensive Cancer Network (NCCN)’s 2010 staging systems [34]. Clinical performance status was assessed using the Eastern Cooperative Oncology Group (ECOG) scale [35] at first-line therapy initiation. Patients were stratified according to the International Metastatic Renal Cell Carcinoma Database Consortium (IMDC) risk classification system [8].

All patients received at least one cycle (4 weeks) of TKIs, continuing until disease progression or loss of therapeutic response. Treatment response was evaluated 3 months after its initiation using the Response Evaluation Criteria in Solid Tumors (RECIST) [36] and Morphology, Attenuation, Size, and Structure (MASS) [37] criteria. Data on second- and third-line treatments were also collected, with treatment responses assessed using the same criteria (detailed in the Section 3).

### 2.2. Immunohistochemistry

Tissue microarrays (TMAs) were constructed using primary mccRCC samples for immunohistochemical (IHC) analysis. IHC was performed on formalin-fixed, paraffin-embedded tumor tissues using a rabbit polyclonal anti-MrgD antibody (reference HPA031346, Sigma-Aldrich, St. Louis, MO, USA, dilution 1:50). The antibody specificity had been previously validated [38]. Staining was performed on an automated immunostainer (Dako Autostainer Plus, Dako-Agilent, Glostrup, Denmark) following standard protocols. Antigen retrieval was carried out in a low-pH buffer (K8005, Dako) at 95 °C for 20 min. Tissue sections were incubated with the primary antibody for 50 min at room temperature, followed by washing and application of a secondary anti-rabbit antibody (K8021, Dako) for 20 min. Detection was achieved using the EnVision-Flex system, which incorporates an HRP-labeled polymer (SM802, Dako). Diaminobenzidine (DAB; DM827, Dako) was used as the chromogen, and sections were counterstained with hematoxylin (K8008, Dako).

Two independent observers evaluated the slides under light microscopy, resolving discrepancies by consensus. Each TMA included two cores per primary tumor. TMAs also included two cores of non-tumor renal tissue as internal controls. In these controls, MrgD staining was categorized as previously reported [32]: moderate or intense in proximal tubules, weak in distal nephrons, and absent in glomeruli. Tumor staining was similarly classified as weak, moderate or intense when ≥10% of tumor cells in a core displayed staining comparable to control tissues. Staining below this threshold (<10%) was considered negative [39]. Following previous classification criteria [32], tumors were grouped into two categories: positive (moderate/strong staining) and negative (weak/absent staining). If one core was negative and the other positive, the case was classified as positive, consistent with the pilot study methodology [32].

### 2.3. Statistical Analysis

Correlations between IHC data and continuous variables (e.g., age, tumor size) were assessed using Spearman’s rank correlation coefficient (Spearman’s Rho test). Categorical variables, including MrgD expression (negative vs. positive), patient sex, pathological characteristics, and treatment response, were analyzed using the Chi-square (χ^2^) test.

Survival analyses were conducted using Kaplan–Meier curves and log-rank tests. Overall survival (OS) was defined as the time from diagnosis to death from any cause, while disease-free survival (DFS) was defined as the time from nephrectomy to the first recurrence or metastasis. To identify independent survival predictors, univariate and multivariate analyses were performed using Cox proportional hazards regression, with the backward Wald elimination method.

All statistical analysis was performed with SPSS^®^ 28.0.

## 3. Results

### 3.1. Patients Clinical and Pathological Characteristics

A total of 170 patients with ccRCC with metastatic disease treated with TKI monotherapy as first-line therapy was initially considered for this study [40]. However, 25 patients were excluded due to undetermined response criteria (n = 5), histological findings inconsistent with clear cell diagnosis (n = 4), or loss to follow-up (n = 16) [33]. Additionally, tissue microarrays from 13 patients were deemed non-representative due to the absence of tumor tissues or core imperfections that precluded pathological evaluation. As a result, this study was conducted with a sample of 132 patients.

In this cohort, the male-to-female ratio was 2.38:1, and the mean age was 59.8 years (median 60 years and range: 26–83 years). The mean tumor size was 8.47 cm (median 8 cm). The Fuhrman grade distribution was as follows: grade 1 in 4 patients (3%), grade 2 in 28 (21.2%), grade 3 in 40 (30.3%), and grade 4 in 60 (45.5%). Regarding the AJCC T category, 18 patients (13.6%) were pT1, 15 (11.4%) were pT2, 90 (68.2%) were pT3, and 9 (6.8%) were pT4. At the time of nephrectomy, the NCCN stage was I in 17 patients (12.9%), II in 8 (6.1%), III in 49 (37.1%), and stage IV in 58 (43.9%). Positive lymph nodes were identified in 20 cases (15.1%), with a single positive node (N1) in 14 patients (10.6%) and multiple positive nodes (N2) in 6 (4.5%). Metastatic disease was present at diagnosis in 51 patients (38.6%), of whom 12 (23.5%) had a single metastasis, and 39 (76.5%) had multiple metastases. Additionally, 81 patients (61.4%) developed metachronous metastases months or years after the initial diagnosis. The mean time from initial diagnosis to nephrectomy was 2.5 months (median: 2 months, range: 1–29 months); from diagnosis to first-line TKI treatment, 29 months (median: 9.5 months, range: 0–201 months); and from nephrectomy to first-line TKI treatment, 28.2 months (median: 9 months, range: 0–201 months).

Performance status was assessed using the ECOG scale, revealing that 111 patients (84.1%) had preserved function (ECOG = 0), 19 (14.4%) had mildly limited function (ECOG = 1), and 2 (1.5%) had moderately limited function (ECOG = 2). Prognostic classification using the IMDC criteria showed that 58 patients (43.9%) had a favorable prognosis, 62 (47%) had an intermediate prognosis, and 12 (9.1%) had an unfavorable prognosis.

This clinical and pathological information is summarized in Table 1, where variables are grouped and dichotomized to facilitate statistical analysis and to ensure consistency with the methodology used in our previous study on early-stage ccRCCs [32]. This approach allows for clearer comparisons and enhances interpretability of the results.

### 3.2. MrgD Expression in Primary Tumor According to Clinical and Pathological Variables

**Figure 1:**
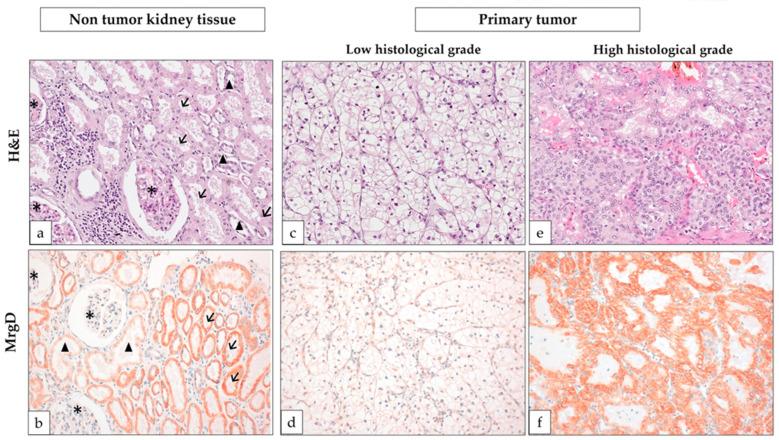
MrgD expression in advanced clear cell renal cell carcinoma (mccRCC). (**a**) Hematoxylin-eosin (H&E) staining of the non-tumor part of the nephrectomized kidney shows proximal convoluted tubules with granular eosinophilic cells (arrows) and glomeruli (asterisks) but also distal nephron tubules (arrowheads). (**b**) MrgD staining of the non-tumor tissue reveals an intense granular cytoplasmic staining of the proximal tubules (arrows) and weak staining of the distal tubules (arrowheads). Notice that glomeruli can be used as internal negative control (asterisk). (**c**,**d**) Low-grade ccRCC shows weak MrgD staining, whereas the high-grade tumor (**e**,**f**) shows intense staining. Original magnification ×250.

Following the same protocol as our previous study [32], we initially evaluated MrgD receptor expression in the non-tumor regions of nephrectomized kidneys. This receptor exhibited variable intensity within the cytoplasm and membranes of the cells along the nephron tubules. Specifically, staining ranged from moderate to intense in proximal tubules and weak to absent in distal nephron tubules, while being entirely absent in glomeruli (Figure 1). Based on these observations in non-tumor tissue, the staining patterns in renal tumors were categorized into two groups: tumors displaying moderate or intense cytoplasmic and membranous staining were classified as positive, whereas those with no staining or only weak staining for MrgD were classified as negative.

#### 3.2.1. MrgD Expression Does Not Change Depending on Patients’ Ages or Sex

The initial statistical analysis revealed no correlation between MrgD expression and the patients’ ages (Spearman rho, r = −0.42, *p* = 0.631). Age was also stratified into a categorical variable based on the median value (60 years). No significant differences in the expression of the protein were observed between patients younger or older than 60 years (χ^2^ test, *p* = 0.68). Similarly, no differences were detected between male and female patients (χ^2^ test, *p* = 0.94).

#### 3.2.2. MrgD Expression Is Higher in High-Grade Primary mccRCCs

**Figure 2:**
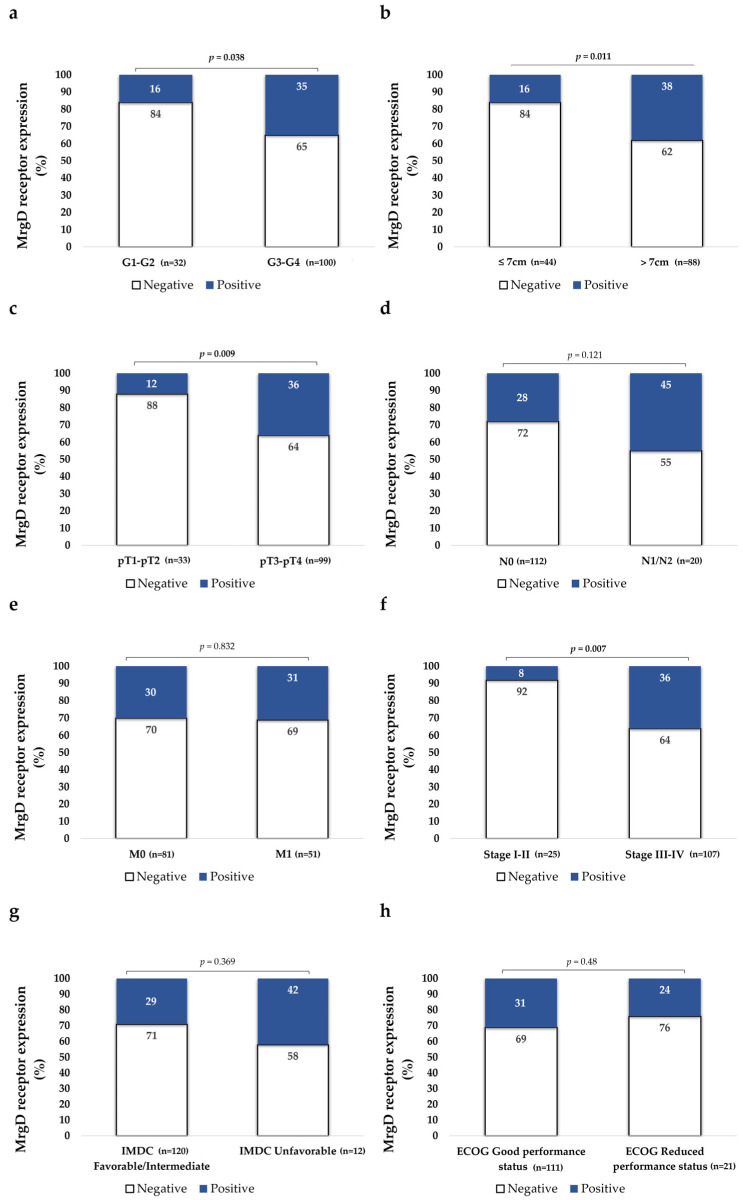
Immunohistochemical staining of MrgD in mccRCC tumors based on clinical and pathological variables grouped according to histological grade (**a**), size (**b**), local invasion (pT) (**c**), lymph node (N) (**d**), distant metastasis (M) (**e**), and NCCN stage at diagnosis (**f**). IMDC risk criteria (**g**) and ECOG performance status (**h**) were evaluated at the time of initiating TKI-therapy. MrgD staining intensity was grouped as negative and positive. The “%” symbol on the Y-axis of the graph represents the percentage of cases that were MrgD positive or negative. The χ^2^ test was used for data analysis. N0: No lymph node metastasis; N1: lymph node metastasis; M0: No distant metastasis; M1: synchronous distant metastasis.

The Fuhrman histological grade of primary tumors was categorized into low (G1–G2) and high grades (G3–G4). MrgD-positive cases were significantly more frequent in high-grade mccRCCs as compared to low-grade ones, as illustrated in Figure 1 and Figure 2.

#### 3.2.3. MrgD Expression Is Higher in Larger and Non-Organ-Confined Tumors

Tumors were classified according to the median sizes into tumors of 7 cm or smaller or those larger than 7 cm, with MrgD staining being significantly more intense in the latter group (Figure 2). Additionally, Spearman’s rho test also demonstrated a positive correlation between MrgD expression and tumor size (r = 0.281, *p* = 0.001).

Results were also stratified according to the local invasion (pT) parameter, and we observed that MrgD expression was significantly more intense in non-organ-confined tumors (pT3–pT4) than in organ-confined ones (pT1–pT2) (Figure 2).

#### 3.2.4. MrgD Staining Is More Intense in Higher-Stage Tumors

There were no differences in MrgD expression between tumors that invaded locoregional lymph nodes and tumors without nodal invasion. Primary tumors from patients diagnosed with synchronous metastasis also had similar MrgD staining intensity than tumors from patients diagnosed with metachronous ones (Figure 2). No significant differences were observed between cases presenting with single or multiple metastases (χ^2^, *p* = 0.38). Tumors were also categorized according to the NCCN 2010 staging system. We observed that stage III-IV tumors showed significantly higher expression of the protein than I-II stage tumors (Figure 2).

#### 3.2.5. MrgD Expression Does Not Change Depending on ECOG Performance Status and IMDC Risk Classification

The expressions of MrgD in primary tumors from patients with mildly to moderately limited functions (ECOG 1–2) were similar to those from patients with preserved functions (ECOG 0). Similarly, the staining intensity of this protein did not vary according to IMDC criteria (Figure 2).

### 3.3. MrgD Expression in Primary Tumor According to Treatment Response

All 132 patients in the cohort received TKI-based first-line therapy with sunitinib, sorafenib, or pazopanib as a monotherapy. Treatment response was assessed using both the MASS and RECIST scoring systems. According to the MASS score, 56 patients (42.4%) exhibited an unfavorable response, 44 (33.3%) demonstrated a favorable response, and 32 (24.2%) had an indeterminate response. RECIST results were similar: 55 patients (41.7%) experienced disease progression, 15 (11.4%) achieved a complete response, 31 (23.5%) had a partial response, and 31 (23.5%) maintained stable disease.

Second-line therapy was administered to 85 patients (64.4%) and included alternative TKIs (n = 52; e.g., axitinib, cabozantinib), mammalian target of rapamycin complex-1 (mTORC1) inhibitors (n = 23; everolimus or temsirolimus), and ICIs (n = 10; nivolumab and/or ipilimumab). Among these patients, 45 (52.9%) had an unfavorable response (MASS) or disease progression (RECIST), 18 (21.2%) exhibited a favorable or partial response, and 22 (25.9%) were classified as having an indeterminate response or stable disease.

Third-line therapy was administered to 29 patients (22%) and consisted of TKI cabozantinib (n = 11), the mTORC1 inhibitor temsirolimus (n = 7), or combinations of ICIs and TKIs (n = 11; pembrolizumab or avelumab plus axitinib). In this group, 14 patients (48.3%) experienced an unfavorable response (MASS) or progression (RECIST), 7 (24.1%) had a favorable or partial response, and 8 (27.6%) were reported as having an indeterminate response or stable disease.

#### 3.3.1. MrgD Expression Associated with Unfavorable Response to TKIs in First-Line Therapy

**Figure 3:**
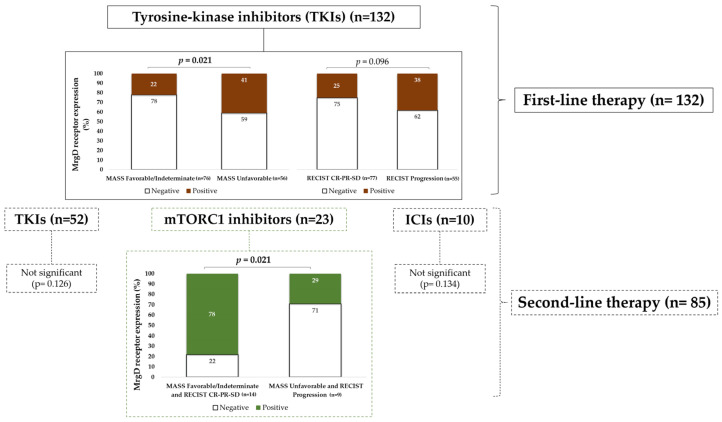
Immunohistochemical staining of MrgD in mccRCC tumors according to patients’ response to first- and second-line therapies. MrgD expression was significantly higher in mccRCCs treated with TKIs as first-line therapy with unfavorable responses (MASS) but lower in cases treated with mTORC1 inhibitors as second-line therapy. The “%” symbol on the Y-axis of the graph represents the percentage of cases that were MrgD positive or negative. CR: Complete response; PR: partial response; SD: stable disease. MrgD staining intensity was grouped as negative or positive. The χ^2^ test was used for data analysis.

Primary tumors from mccRCC patients with an unfavorable response (MASS) to sunitinib, pazopanib, or sorafenib exhibited significantly higher MrgD staining intensities as compared to tumors with favorable or indeterminate outcomes. A similar trend was observed when the data were stratified using RECIST criteria; however, the differences did not reach statistical significance (Figure 3).

#### 3.3.2. MrgD Expression Was Lower in Tumors with Unfavorable Responses to mTORC1 Inhibitors in Second-Line Therapy

Outcomes after second-line therapy were stratified in two ways. First, the analysis was performed on the entire cohort (n = 85) as a single group. No significant differences in MrgD expression were observed when stratified by either RECIST or MASS criteria (χ^2^, *p* = 0.912 for both MASS and RECIST).

The second approach involved stratification by drug class (Figure 3), and significant results were observed in the subset of patients receiving mTORC1 inhibitors (n = 23). Tumors from patients with unfavorable responses and disease progression (n = 14 in both cases) showed lower MrgD expressions as compared to tumors with favorable, partial, indeterminate, or stable responses (n = 9).

Second-line therapy with axitinib or cabozantinib (n = 52) showed a similar trend to first-line therapy. mccRCCs with unfavorable response and disease progression (n = 29) showed higher MrgD staining intensity than tumors with favorable, partial, indeterminate, or stable responses (n = 23). However, this result did not reach statistical significance (χ^2^, *p* = 0.126 for both MASS and RECIST). The sample size of patients receiving ICIs was too low (n = 10) and heterogeneously distributed between the two groups (poor response, n = 1; rest, n = 9) to make reliable comparisons (χ^2^, *p* = 0.134 for both MASS and RECIST).

For third-line therapy, the analysis was limited to the entire cohort (n = 29) due to the small sample size, which precluded stratification by drug class. No significant differences in MrgD expression were observed between tumors with unfavorable responses or disease progression and other outcomes (χ^2^, *p* = 0.584 for both MASS and RECIST).

### 3.4. MrgD Expression in mccRCC According to Patients’ Survival

#### 3.4.1. MrgD Expression Is Associated with Worse Overall Survival (OS) for mccRCC Patients

OS was defined as the time from diagnosis to death from any cause [41]. The mean follow-up time for the 132 patients in the series was 66.4 months (median: 50 months), ranging from a minimum of 3 months to a maximum of 247 months. We performed OS analyses at different follow-up intervals, spanning from 1 to 15 years, as detailed in Table 2. By the 15-year mark, 124 patients (93.9%) had died, while 8 (6.1%) were still alive.

**Figure 4:**
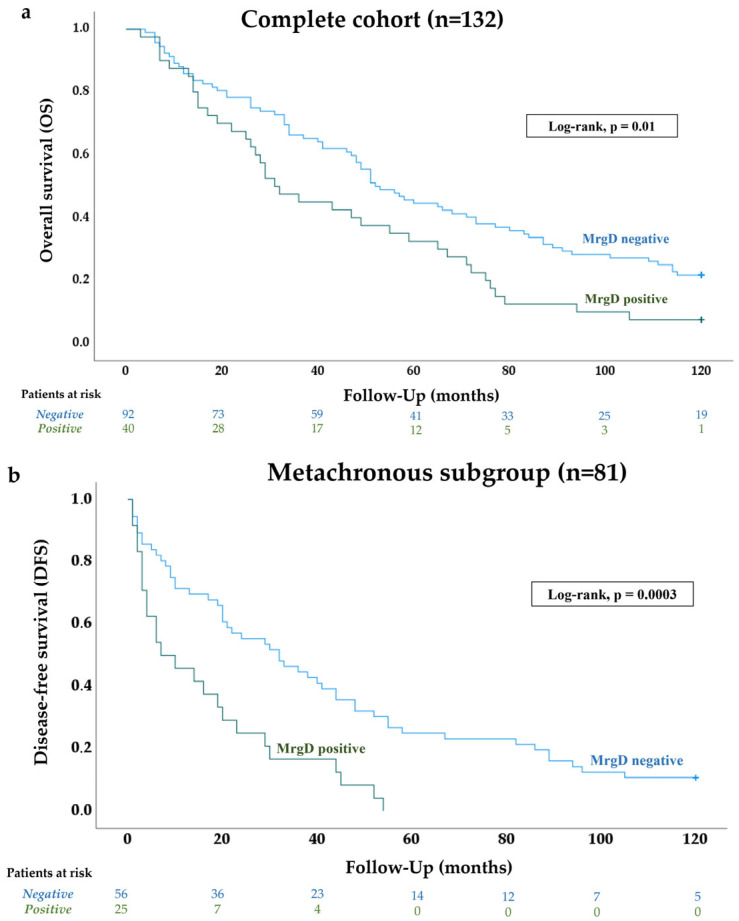
MrgD expression in mccRCC and patients’ survival. Kaplan–Meier curves and log–rank test demonstrated the significant association between MrgD positivity and 10-year overall (**a**) and disease-free (**b**) survival. The number of patients at risk is shown at specific time points throughout the follow-up period.

Figure 4 presents the Kaplan–Meier curve for 10-year overall survival (OS) in mccRCC patients. MrgD positivity was significantly associated with poorer OS at 3, 4, 8, 10, and 15 years of follow-up (Table 2).

Univariate Cox regression model analysis was performed to evaluate the correlations between MrgD expression and pathological variables with 10-year OS (Table 3). The data demonstrated that age (cutoff = 60 years), grouped grade, pT stage, lymph node invasion (N), distant metastasis at diagnosis (M), ECOG score, IMDC risk group, MASS score, RECIST response after first-line therapy, and MrgD expression were significantly associated with poorer OS. These variables were subsequently included in the multivariate Cox regression model.

To minimize mathematical bias arising from collinearity between variables, tumor diameter was omitted from the analysis because local invasion (pT) encompasses this variable. Similarly, NCCN 2010 Stage was excluded as it is composed of pT, N, and M. By multivariate Cox regression model analysis, built using a backward Wald stepwise elimination approach, age, grouped grade, pT stage, M, IMDC risk group, and MASS score were all identified as independent prognostic factors of 10-year OS (Table 3).

Additionally, univariate and multivariate analyses were conducted for 5- and 15-year OS data (Supplementary Table S1). For 5 years, the OS grouped grade, M, IMDC risk group, and RECIST response were found to be independent variables. For 15 years, the OS age, grouped grade, pT stage, M, IMDC risk group, and MASS score were identified as independent variables. However, we did not observe that MrgD expression was associated with OS in these analyses.

#### 3.4.2. MrgD Expression Is Associated with Worse Disease-Free Survival (DFS) for Patients with Metachronous Metastases

DFS reflects the duration of remission after initial curative-intent surgery [42]. In this cohort, it was calculated based on the time between nephrectomy and the initiation of first-line TKI treatment, as all patients received targeted therapy shortly after recurrence was diagnosed. In the group of 81 patients (61.4%) who did not present distant metastases at the time of initial diagnosis, the nephrectomy was apparently curative. However, months or years later, all these patients developed metachronous metastases. On average, metastases were diagnosed at 39 months (median: 21.5, range: 1 to 201 months), and by the 15-year follow-up, 79 patients (97.5%) had relapsed.

DFS was analyzed across various follow-up intervals, ranging from 1 to 15 years. As shown in Table 2, MrgD expression in primary tumors was significantly associated with DFS at all follow-up time points. Figure 4 presents the representative Kaplan–Meier curve for 10-year DFS.

Univariate analysis was conducted at 10-year DFS (Table 3) but also at 5- and 15-year DFS (Supplementary Table S1). Variables such as metastasis and NCCN 2010 stage were excluded, as the cohort consisted exclusively of metachronous cases (M = 0) and due to the overlap with pT and N categories. MASS and RECIST scores were also omitted, as these metrics assess treatment response, and this subgroup of patients remained treatment-naïve until the development of metastatic disease. Similarly, ECOG and IMDC scales were excluded, as these assessments were performed at the time of disease recurrence and the initiation of systemic therapy.

Age (cutoff: 60 years), grouped grade, pT and N categories, and MrgD expression were individually associated with 10-year DFS and were included in the multivariate analysis. Using a backward Wald stepwise-elimination approach, MrgD was identified as an independent prognostic factor for 10-year DFS alongside age, tumor grade, and pT stage (Table 3). Similar analyses at 5 and 15 years also confirmed MrgD as an independent biomarker predicting DFS (Supplementary Table S1).

## 4. Discussion

This study characterizes MrgD expression in primary tumors from patients with mccRCC who underwent sequential treatment with TKIs as the first-line therapy. The findings extend our previous investigation of MrgD expression in early-stage disease [32], confirming and expanding upon earlier observations and underscoring the prognostic and therapeutic significance of this protein in ccRCC.

Before the validation in tumor tissues, we aimed to confirm the staining pattern in non-tumor kidney tissue [32,38], and indeed, the analysis corroborated that MrgD is present in the cytoplasm and membranes of tubular cells. The expression was high in proximal tubules, which are the sites of origin for ccRCC according to the most widely accepted theory [1,3].

The stratification of MrgD expression by pathological variables showed that tumors with higher histological grades, larger sizes, and advanced pathological stages exhibited significantly higher staining intensity of this protein. Furthermore, patients’ OS was worse when this receptor was highly expressed at the primary tumor. These results mirror those from the previous study [32], reinforcing the hypothesis that MrgD expression correlates with the intrinsic biological aggressiveness of ccRCC across disease stages.

A subset of 81 patients in the cohort developed metastases months or years after initial diagnosis, enabling an analysis of the association between MrgD expression and DFS. During a follow-up period of up to 15 years, higher MrgD expression was independently associated with a shorter time of recurrence. This finding adds substantial value to our previous pilot study of early-stage ccRCC [32], as it positions this receptor as a biomarker for identifying patients at greater risk of metachronous metastasis.

The identification of reliable IHC biomarkers for ccRCC remains challenging due to the marked intratumor heterogeneity of this disease [43]. However, prior studies demonstrated that MrgD [32], alongside other components of the intrarenal RAS [44], exhibits a homogeneous spatial distribution across the tumor center and periphery. This uniformity enhances its reliability and potential utility as a robust prognostic biomarker for routine clinical practice. Nonetheless, validation in larger and multicenter studies is necessary to confirm this potential.

ccRCC is a good example of a highly vascularized solid tumor [3]. In cancer, there is a strong association between vascular endothelial growth factor (VEGF)-dependent neoangiogenesis and immunosuppressive microenvironments [45]. This relationship underpins the significant therapeutic advances achieved with the combination of TKIs and ICIs in advanced ccRCC [6,7].

Components of RAS have been identified in tumor cells but also in endothelial, immune, and other stromal cells [13,45,46]. The expressions of AT1R in tumor cells [20] and ACE in tumor vessels [22] are associated with aggressive behavior in ccRCC. Additionally, Ang-II/AT1R signaling pathways induce VEGF receptor-mediated angiogenesis and promote desmoplastic tumor microenvironments that impair immune responses [46]. Furthermore, they stimulate the release of various pro-inflammatory cytokines by both tumor and stromal cells, which collectively suppress the action of immunostimulatory cells while activating the recruitment and function of tumor-promoting immune cells [46,47].

These cancer hallmarks, driven by conventional RAS, are linked to the activation of signaling pathways such as PI3K/AKT/mTORC1 [13]. Inhibiting this pathway at various levels forms the basis of therapeutic strategies for advanced ccRCC, including TKIs and mTORC1 inhibitors [5]. Furthermore, RAS-induced tumor-promoting phenomena are reversed by RASis [46,47], providing a mechanistic explanation for the enhanced efficacy of targeted therapies and ICIs in mccRCC patients who concomitantly use ARBs or ACEis [11,12,15,16].

The alternative RAS exerts counter-regulatory effects in processes such as proliferation and inflammation, primarily by inhibiting signaling pathways activated by the conventional RAS, including the PI3K/AKT/mTORC1 pathway [13]. Through this mechanism, the ALA/MrgD axis suppresses the secretion of pro-inflammatory cytokines in the kidney [48] and inhibits proliferation in pancreatic cancer cell lines [31]. However, this counter-regulatory role does not appear to apply universally, as tumor cell growth is promoted by activation of ALA in non-small-cell lung cancers, where MrgD is highly expressed [29,30]. This finding aligns with our results for ccRCC and suggests that the role of this receptor in cancer is highly tumor-specific.

Studies on the ACE2/Ang 1-7/MAS axis in renal cancer also yield conflicting results, depending on the cell lines and xenograft models analyzed. Findings range from tumor growth inhibition and improved TKI efficacy [24] to stimulation of invasive behaviors [23,25]. Furthermore, studies in tumor tissues reveal that while higher *ACE2* mRNA levels are associated with increased survival in ccRCC patients [24], IHC expression of the protein correlates with more aggressive ccRCCs [22]. Together, these findings highlight the complex role of the alternative RAS in the specific context of renal cancer.

Similarly, our study also revealed a complex and drug-class-dependent relationship between MrgD expression and therapeutic outcomes in mccRCC. High staining intensity of this receptor was associated with poorer responses to TKIs in the first-line setting, with a similar trend observed for other TKIs in second-line treatment (although this did not reach statistical significance). Conversely, patients receiving the mTORC1 inhibitors as second-line therapy exhibited significantly better responses when MrgD expression in the primary tumor was higher.

Given that both drug classes inhibit the PI3K/AKT/mTORC1 pathway at different levels [5], forming hypotheses to explain this result is challenging. Improved responses to mTORC1 inhibitors in mccRCCs with high MrgD expression could stem from a synergistic effect between these drugs and the downstream inhibitory signaling of this pathway triggered by MrgD stimulation [13]. However, such synergy would also be expected with TKIs, yet the data suggest opposing effects. This finding warrants further investigation to explore the potential of MrgD as a predictive biomarker for treatment selection, offering a renewed role for therapies currently relegated to third-line use in advanced ccRCC protocols [5,6]. This cohort allows the investigation of the role of MrgD as a potential prognostic biomarker, both for OS and DFS. However, a limitation of this study in evaluating treatment response is the time gap between the immunohistochemical staining of MrgD in primary ccRCC tissues from nephrectomy and the start of first-line treatment. It can be expected that the intimate relation between the treatment response and the tumor marker should be better evaluated in metastatic tissue at the time of initiating treatment.

Future studies should address several critical aspects. First, they should encompass large patient cohorts, incorporating the latest therapeutic protocols combining ICIs and TKIs with extended follow-up periods [5,6]. Additionally, the inclusion of metastatic tissues is essential to validate treatment response findings observed in primary tumors. Studies in cell lines and animal models will also be instrumental in elucidating the role of the ALA/MrgD axis in renal carcinogenesis. As part of these experimental approaches, it will also be important to investigate the observed mRNA–protein discrepancies for MrgD [38] to explore potential post-transcriptional or post-translational regulatory mechanisms, as previously described for other components of RAS in both neoplastic and non-neoplastic kidney diseases [21,49]. These models may further clarify whether the receptor requires heteromerization with MASR to mediate intracellular signaling, as recently described in inflammatory contexts involving macrophages [50]. Additionally, they could help determine whether MrgD possesses intrinsic constitutive activity in tumors indicative of RAS-independent mechanisms, similar to observations in renal (HEK293) [51] and tumor (HeLa) cell lines [52].

## 5. Conclusions

This study validates and expands the prognostic relevance of MrgD expression in ccRCC, confirming its consistent association with tumor aggressiveness and risk of recurrence. Additionally, our findings reinforce the association between MrgD expression and differential therapeutic responses in advanced disease, as observed in our previous study. Future research integrating mechanistic, preclinical, and clinical studies will be critical to fully realize the translational potential of this novel receptor.

## Figures and Tables

**Table 1:** Pathological and clinical characteristics of patients at initial diagnosis and at TKI start *.

Variables	n = 132
**Age**	
≤60 years	69
>60 years	63
**Gender**	
Male	93
Female	39
**Fuhrman grade**	
Low (G1–G2)	32
High (G3–G4)	100
**Diameter**	
≤7 cm	44
>7 cm	88
**Local invasion (pT)**	
Organ-confined (pT1–pT2)	33
Not confined (pT3–pT4)	99
**Lymph node invasion (N)**	
No	112
Yes	20
**Metastasis (M)**	
Metachronous	81
Synchronous	51
**NCCN 2010 Stage**	
Stage I-II	25
Stage III-IV	107
ECOG ^(^*^)^	
Preserved function (ECOG 0)	111
Reduced function (ECOG 1-2)	21
IMDC ^(^*^)^	
Favorable/Intermediate	120
Unfavorable	12

NCCN: National Comprehensive Cancer Network; ECOG: Eastern Cooperative Oncology Group; IMDC: International Metastatic Renal Cell Carcinoma Database Consortium.

**Table 2:** Overall (OS) and Disease-Free (DFS) survival depending on MrgD expression. Log–rank test was used to analyze the association between MrgD and patients’ survival.

**OS**	1 year	2 years	3 years	4 years	5 years	8 years	10 years	15 years
Log-rank *p*=	0.83	0.21	**0.02**	**0.04**	0.09	**0.009**	**0.01**	**0.003**
**DFS**	1 year	2 years	3 years	4 years	5 years	8 years	10 years	15 years
Log-rank *p*=	**0.01**	**0.004**	**0.003**	**0.002**	**0.0009**	**0.0003**	**0.0003**	**0.0003**

**Table 3:** Predictive model (Cox regression) for 10-year overall survival (OS) and disease-free survival (DFS) prediction by MrgD and pathological variables in mccRCC patients. Selected independent variables included MrgD expression (negative vs. positive), clinical, pathological, and treatment response variables, which were grouped and dichotomized as described in Table 1 and Figure 2 and Figure 4. Hazard Ratios (HR) with Confidence Intervals (CI) are also included. Variables resulting from the backward Wald stepwise method are highlighted in bold.

Cox Regression Model		10-Year Overall Survival			10-Year Disease-Free Survival		
	Variables	*p*	HR	95% CI	*p*	HR	95% CI
Univariate analysis	Sex	0.22	0.78	0.52–1.16	0.7	1.11	0.66–1.86
	Age	0.007	1.66	1.15–2.4	0.049	1.58	1–2.5
	Grade	0.002	1.99	1.28–3.1	0.001	2.34	1.42–3.86
	pT	<0.001	2.26	1.43–3.55	0.001	2.32	1.38–3.89
	N	<0.001	2.86	1.79–4.57	0.003	3.12	1.46–6.69
	M	<0.001	3.12	2.14–4.55	-	-	-
	ECOG	0.005	1.91	1.21–3	-	-	-
	IMDC	<0.001	4.15	2.35–7.33	-	-	-
	MASS	<0.001	2.98	2.06–4.32	-	-	-
	RECIST	<0.001	3.23	2.23–4.69	-	-	-
	MrgD	0.011	1.68	1.12–2.51	0.001	2.49	1.48–4.2
Multivariate analysisFinal step of Wald method	Age	**0.013**	1.66	1.11–2.48	**0.001**	2.56	1.53–4.28
	Grade	0.038	1.68	1.03–2.75	**0.001**	2.54	1.45–4.44
	pT	0.05	1.7	1–2.89	**0.011**	2.14	1.19–3.86
	M	**0.001**	2.28	1.51–3.45	-	-	-
	IMDC	0.003	2.7	1.42–5.14	-	-	-
	MASS	0.001	3.24	2.15–4.88	-	-	-
	MrgD	-	-	-	**0.049**	1.76	1–3.03

## Data Availability

Anonymized datasets used and/or analyzed during the current study are available from the corresponding author upon reasonable request.

## Supplementary Materials

The following supporting information can be downloaded at https://www.mdpi.com/article/10.3390/biom15030387/s1: Supplementary Table S1: Predictive model (Cox regression) for 5- and 15-year overall (OS) and disease-free survival (DFS) prediction by MrgD and pathological variables in mCCRCC patients.
