# Supplementary material for: Prognostic and Therapeutic Implications of Alamandine Receptor MrgD Expression in Clear Cell Renal Cell Carcinoma with Development of Metastatic Disease
Source: Biomolecules. 2025 Mar 7;15(3):387. doi: 10.3390/biom15030387 (PMC11939982; doi:10.3390/biom15030387)
Supplement: Supplementary file 1 [file biomolecules-15-00387-s001.zip › biomolecules-3452407-supplementary.pdf]

Supplementary Table S1. Predictive model (Cox regression) for 5- and 15-year overall (OS) and disease-free survival (DFS) prediction by MrgD and pathological variables in mCCRCC patients. Selected independent variables included MrgD expression, clinical, pathological, and treatment response variables, which were grouped and dichotomized as described in Table 1 and Figures 2 and 4. Hazard Ratios (HR) with Confidence Intervals (CI) are also included. Variables resulting from the backward Wald stepwise method are highlighted in bold.

A) 5-year OS and DFS

Cox Regression Model		5-year OS				5-year DFS		
	**Variables**	**p**	**HR**	**CI**		**p**	**HR**	**CI**
Univariate analysis	Sex	0.063	0.65	0.42-1.02		0.885	1.04	0.6-1.8
	Age	0.065	1.49	0.98-2.27		0.03	1.7	1.05-2.74
	Grade	0.003	2.34	1.34-4.09		0.002	2.3	1.34-3.93
	pT	0.019	1.89	1.11-3.21		0.01	2.04	1.19-3.5
	N	< 0.001	2.71	1.64-4.49		0.003	3.12	1.46-6.69
	M	< 0.001	3.65	2.37-5.64		-	-	-
	ECOG	0.007	1.9	1.2-3.27		-	-	-
	IMDC	< 0.001	4.15	2.35-7.33		-	-	-
	MASS	< 0.001	3.86	2.5-5.98		-	-	-
	RECIST	< 0.001	3.95	2.54-6.12		-	-	-
	MrgD	0.096	1.49	0.93-2.37		0.002	2.34	1.38-3.95
Multivariate analysis<br>Final step of Wald method	Age	-	-	-	-	**0.001**	2.64	1.53-4.55
	Grade	**0.029**	1.98	1.07	3.64	**0.003**	2.57	1.39-4.78
	pT	-	-	-	-	-	-	-
	N	-	-	-	-	0.067	2.08	0.95-4.55
	M	**0.001**	2.54	1.59	4.05	-	-	-
	IMDC	0.001	3.88	1.97	7.63	-	-	-
	RECIST	0.001	4.46	2.73	7.28	-	-	-
	MrgD	-	-	-	-	**0.043**	1.77	1.02-3.07

B) 15-year OS and DFS

Cox Regression Model	Variables	15-year OS				15-year DFS		
		p	HR	CI		p	HR	CI
Univariate analysis	Sex	0.425	0.86	0.59-1.25		0.66	1.11	0.67-1.86
	Age	0.002	1.77	1.24-2.51		0.03	1.65	1.05-2.6
	Grade	< 0.001	2.07	1.37-3.11		0.001	2.33	1.44-3.79
	pT	< 0.001	2.1	1.38-3.08		0.003	2.1	1.29-3.47
	N	< 0.001	2.86	1.79-4.57		0.003	3.12	1.46-6.69
	M	< 0.001	3.06	2.13-4.38		-	-	-
	ECOG	0.01	1.78	1.15-2.75		-	-	-
	IMDC	< 0.001	4.15	2.35-7.33		-	-	-
	MASS	< 0.001	2.67	1.88-3.78		-	-	-
	RECIST	< 0.001	2.67	1.88-3.78		-	-	-
	MrgD	0.004	1.76	1.2-2.6		<0.001	2.49	1.48-4.18
Multivariate analysis<br>Final step of Wald method	Age	**0.001**	1.89	1.29	2.78	**0.001**	2.66	1.6-4.43
	Grade	**0.008**	1.86	1.18	2.94	**0.001**	1.98	1.13-3.48
	pT	0.063	1.55	0.98	2.46	**0.017**	1.98	1.13-3.48
	M	**0.001**	2.28	1.54	3.39	-	-	-
	IMDC	**0.004**	2.57	1.35	4.79	-	-	-
	MASS	0.001	2.84	1.93	4.19	-	-	-
	MrgD	-	-	-	-	**0.042**	1.75	1.02-3.02
